# *Fusarium solani* infection disrupts metabolism during the germination of roselle (*Hibiscus sabdariffa* L.) seeds

**DOI:** 10.3389/fpls.2023.1225426

**Published:** 2023-08-08

**Authors:** Aminallah Tahmasebi, Thomas Roach, Song Yub Shin, Chul Won Lee

**Affiliations:** ^1^ Department of Agriculture, Minab Higher Education Center, University of Hormozgan, Bandar Abbas, Iran; ^2^ Department of Chemistry, Chonnam National University, Gwangju, Republic of Korea; ^3^ Department of Botany, University of Innsbruck, Innsbruck, Austria; ^4^ Graduate School of Biomedical Science, Department of Cellular & Molecular Medicine, School of Medicine, Chosun University, Gwangju, Republic of Korea

**Keywords:** biochemical components, energy reserves, *Fusarium solani*, *Hibiscus sabdariffa*, seed quality

## Abstract

Fungal infections adversely influence the production and quality of seeds. Previously, *Fusarium solani* was reported as the causal agent of roselle (*Hibiscus sabdariffa* L.) seed rot. This study was designed to evaluate the effect of *F. solani* infection on the germination, biochemical composition, energy reserves, and antioxidant activity of roselle seeds because there is currently a lack of information on the relationship between seed metabolism and infection with *F. solani*. The results showed that roselle seeds infected with *F. solani* exhibited a ca. 55% reduction in overall germination. Additionally, the fungal infection decreased antioxidant activity, total phenolic content, protein, sugar (sucrose, fructose, and glucose), and some amino acid (glutamine, serine, and arginine) contents. In contrast, some metabolites were more abundant in infected seeds, including alanine (2.1-fold) and some fatty acids (palmitic acid and heptadecanoic acid by 1.1- and 1.4-fold, respectively). The infection-associated changes in fatty acid profile resulted in the ratio of unsaturated/saturated fatty acids being 2.1-fold higher in infected seeds. Therefore, our results reveal that *F. solani* infection remarkably altered the biochemical composition of roselle seeds, which may have contributed to the loss of germination and quality of roselle seeds.

## 1 Introduction

The tropical plant roselle (*Hibiscus sabdariffa*) belongs to the Malvaceae family and is used for a variety of therapeutic purposes in traditional medicine (Nyam et al., 2014), in agreement with its exhibiting a variety of medicinal and nutritional properties (Riaz and Chopra, 2018). In Africa, roselle seeds are ground into meals for human consumption and also roasted as a coffee alternative (Mohd-Esa et al., 2010). A great source of nutritional fiber is also found in roselle seeds, and the addition of roselle seed powder to cookies enhances their antioxidant capabilities (Nyam et al., 2014). Furthermore, it has also been shown that roselle seeds contain rich sources of antioxidants, proteins, carbohydrates, and fatty acids, which may be employed in the food industry (Mohd-Esa et al., 2010; Cissouma et al., 2013; Peng et al., 2020).

Seeds are one of the most significant sources of food with vital nutrients such as lipids, carbohydrates, and proteins (Aguirre et al., 2018). Roselle seeds maintain a significant amount (ca. 17% of dry weight) of storage reserves as oil (Mohamed et al., 2007). Further to their use as energy reserves, fatty acids are utilized during plant defense against pathogens (e.g., for the production of jasmonates), seed germination, and seedling establishment (Kachroo and Kachroo, 2009; Chen et al., 2012; Shrestha et al., 2016). Phenolic compounds also play vital roles in seed germination, development, and resistance against biotic and abiotic stresses (Corso et al., 2020). Seed storage proteins are considered storage reserves for sulfur, carbon, and nitrogen for germinating seedlings (Krishnan and Coe, 2001). Amino acids can contribute to seed energy reserves and provide precursors for protein synthesis and the production of secondary metabolites (Amir et al., 2018). Carbohydrates are also one of the key energy sources and play a significant role in seed development (Aguirre et al., 2018).

A wide number of plant pathogens infect roselle plants, weakening the plants in the field and spoiling seeds during storage, resulting in a reduction in seed yield and seedling vigor, respectively (Mcclintock and El-Tahir, 2004; Goko et al., 2021). It has been estimated that seed diseases cause severe losses (more than 40%) of harvested seeds (Tsai and Ou, 1996). Moreover, infected seeds could disseminate primary fungal infection sources to new hosts and locations (Agarval and Sinclair, 1987). It has also been demonstrated that mold infections decrease the nutrient content of seeds (Jun-Cai et al., 2015; Deng et al., 2022). Among fungi, *Fusarium* species were found to infect and economically damage a wide number of plants (Dusengemungu, 2021; Wang et al., 2021). It has also been shown that *Fusarium solani* is the major pathogen in roselle that could cause wilt disease (Hassan et al., 2014; Wang et al., 2021). Previously, *F. solani* was shown to be the causal agent of roselle seed rot in Iran (Tahmasebi et al., 2023). Moreover, to the best of our knowledge, there is no report on the effect of fungal infection on the biochemical composition and germination of roselle seeds. Therefore, this study investigated how *F. solani* influences seed germination. We hypothesized that fungal infections disrupt seed metabolism associated with energy reserves and antioxidant activity of roselle seeds, which may help partially explain the influence of the fungi on seed germination.

## 2 Materials and methods

### 2.1 Plant materials and treatments

Roselle seeds were harvested from a research field (longitude and latitude of 57.092371 and 27.091311 and 32 m elevation) at the Minab Higher Education Center, Iran, in April 2021 and kept at 4°C. The average temperature of the location was between 11°C and 39°C. Intact seeds were weighed, and the seeds with similar weight and size were selected. The surface of roselle seeds was sterilized with sodium hypochlorite (1%) solution for 10 min and then rinsed with sterile distilled water. Based on morphological and molecular traits, *F. solani* was previously determined as the causal agent of roselle seed rot (Tahmasebi et al., 2023). Treatments were divided into two groups under the same conditions: *F. solani*-infected (infected) and control (not treated with the fungus) seeds. For infection, dry seeds were inoculated with a 10-day pure culture of *F. solani*. Control and fungus-coated seeds were kept on sterile filter paper in Petri dishes and incubated at room temperature. For infected seeds, fungal mycelium could be used to cover the roselle seeds. The germination percentage of both treatments was checked daily and ended when no more seeds had germinated for three consecutive days. The seeds were considered to have germinated when the radicles reached a length of >2 mm. The experiment was performed with three biological replications, each containing 12 seeds.

### 2.2 Preparation of extracts

*F. solani*-infected and control seeds were freeze-dried (Dena Vacuum Industry Co., 5005, Tehran, Iran) and then ground with mortar and pestle. The samples were stored at −20°C for further analysis.

### 2.3 Antioxidant activity

Powdered seed samples were extracted with 80% (v/v) methanol for 1 h at room temperature using a shaker (KS 260 basic, IKA, Janke & Kunkel, Staufen, Germany). The ratio of seed material to 80% methanol was 0.1% (w/v) and filtered through Whatman filter paper before measurement. For measurement, 1 mL of 1 mM 2,2-diphenyl-1-picrylhydrazyl (DPPH) dissolved in 80% (v/v) methanol was added to 5 mL of seed extract. For control, 5 mL of 80% methanol was used instead of a seed extract. All samples were incubated at room temperature in the dark for 30 min, and their absorbance values were measured at 517 nm (Mohd-Esa et al., 2010) using the spectrophotometer (DR3900, Hach, Germany). The percentage of antioxidant activity was computed according to the formula.


Percentage of   antioxidant activity (%) was calculated as=   [(Ac −As)÷Ac] × 100

whereby Ac represents control absorbance and As shows sample absorbance.

### 2.4 Measurement of total phenolic content

Total phenolic content was determined with Folin–Ciocalteu assay (Mohd-Esa et al., 2010) in 0.2 mL of each sample extract (1 mg mL^−1^ in 80% methanol) with 1 mL of a 10-fold dilution of Folin–Ciocalteu reagent and 0.8 mL of bicarbonate solution (7.5% (w/v)). The mixtures were kept at room temperature for 30 min, and their absorbance was checked at 765 nm using a spectrophotometer (DR3900, Hach Lange, Düsseldorf, Germany). The standard curve was obtained using the preparation of gallic acid concentrations (0.00625, 0.0125, 0.025, 0.05, and 0.1 mg mL^−1^) in 80% methanol.

### 2.5 Total protein assay

For each sample, ground seed samples were homogenized (1:10) in the extraction buffer (50 mM Tris HCl [pH 8] and 2-mercaptoethanol (2%). The homogenates were vortexed, shaken for 1 h, and centrifuged (PIT320 R, Pole Ideal Tajhiz Co., Tehran, Iran) for 5 min at 12,000 rpm at 4°C. The Bradford reagent was combined with 0.1 mL of supernatant. The homogenates were vortexed and kept at room temperature for 10 min, before measuring absorbance at 595 nm using a spectrophotometer (DR3900, Hach, Germany) and quantified against a standard curve of bovine serum albumin concentrations (50, 100, 200, 300, and 400 µg mL^−1^).

### 2.6 Free amino acid analysis by LC-MS/MS

For each sample, 2 g of seed powder was homogenized with 25 mL of 0.1 N hydrochloric acid solution (HCl). The extract was centrifuged at 13,000 rpm for 15 min at 4°C. LC-MS/MS analysis was performed on a Waters TQ-MS (Singapore) system. Chromatography was performed using a C18 column (Kinetex 1.7 µm, 2.1 mm × 100 mm) at a temperature of 38°C with 5 mM ammonium acetate (mobile phase A) and acetonitrile with 0.1% formic acid and 95% methanol in water with 0.1% formic acid (mobile phase B), at a rate of 0.3 mL min^−1^. The sample injection volume was 3 μL. The separation was achieved with a total analysis time of 20 min. Amino acid concentrations were calculated according to the direct comparison of analyte peak areas relative to the standards. The mixture of 17 amino acid standard solution (Supelco, Bellefonte, PA, USA) was used in this study.

### 2.7 Fatty acid analysis by GC-MS

For each sample, 500 mg of ground seed powder was treated with 10 mL of methanol/acetyl chloride (95:5, v/v) solution. The mixture was heated at 80°C for 1 h, then cooled, and 5 mL of double-distilled water was added. The mixture was thoroughly shaken for 5 min. The mixture received 1 mL of hexane and was shaken for 5 min. Afterward, 1 mL of hexane was added again to the mixture before centrifugation at 5,000×*g* at room temperature for 5 min. The supernatant (hexane layer) contained fatty acid methyl esters (FAMES), of which 1 µL was injected with the split ratio (1:8). The GC system (Beifen, 3420A, Beijing, China) was outfitted with a flame ionization detector, a split/splitless injector, and a BPX70 *bis*-cyanopropyl siloxane-sil phenylene capillary column (30 m × 0.25 mm internal diameter and 0.25 µm film thickness) to examine the fatty acid composition. After holding the column temperature at 140°C for 5 min, it was raised to 180°C at a rate of 20°C min^−1^. Following a 9-min hold at 180°C, the temperature was increased to 200°C at a 20°C min^−1^ rate. The temperature of 200°C was maintained for 3 min. The injector and detector were set to operate at 250°C and 300°C, respectively. The carrier gas used was nitrogen. Fatty acid standards processed and injected under the same operating circumstances were used to identify the fatty acids in the samples. Individual FAMEs were quantitatively calculated by determining their relative peak area of total peak areas.

### 2.8 Sugar (sucrose, fructose, and glucose) analysis by HPLC

For each sample, 1 g was homogenized with 15 mL of distilled water and then placed in ultrasonic bath water (Ultrasonic Bath DT 31 H, 35 kHz, BANDELIN Electronic, Berlin, Germany) at 35°C for 15 min and vortexed every 5 min. The homogenate was centrifuged at 10,000 rpm for 5 min, and the supernatant was filtered using a 0.45-μm-pore-size filter. Identification and quantification of sugars were conducted through an HPLC system (Knauer, Berlin, Germany) comprising a P1000 pump and a refractive index (RI) 2300 detector to monitor eluted soluble sugars. Seed extracts (20 μL) were injected into a Eurokat PB column with an inner diameter of 300 mm × 10 mm and a particle size of 10 μm (Knauer, Germany). The mobile phase was HPLC-grade water with 1 mL min^−1^ flow rate at 65°C.

### 2.9 Statistical analysis

An independent sample *t*-test was performed to compare significant differences between two treatments (infected and noninfected seeds) using SPSS (version 21) at *p*< 0.05 significance level (two-tailed).

## 3 Results and discussion

### 3.1 The influence of fungal infection on seed germination

**Figure 1:**
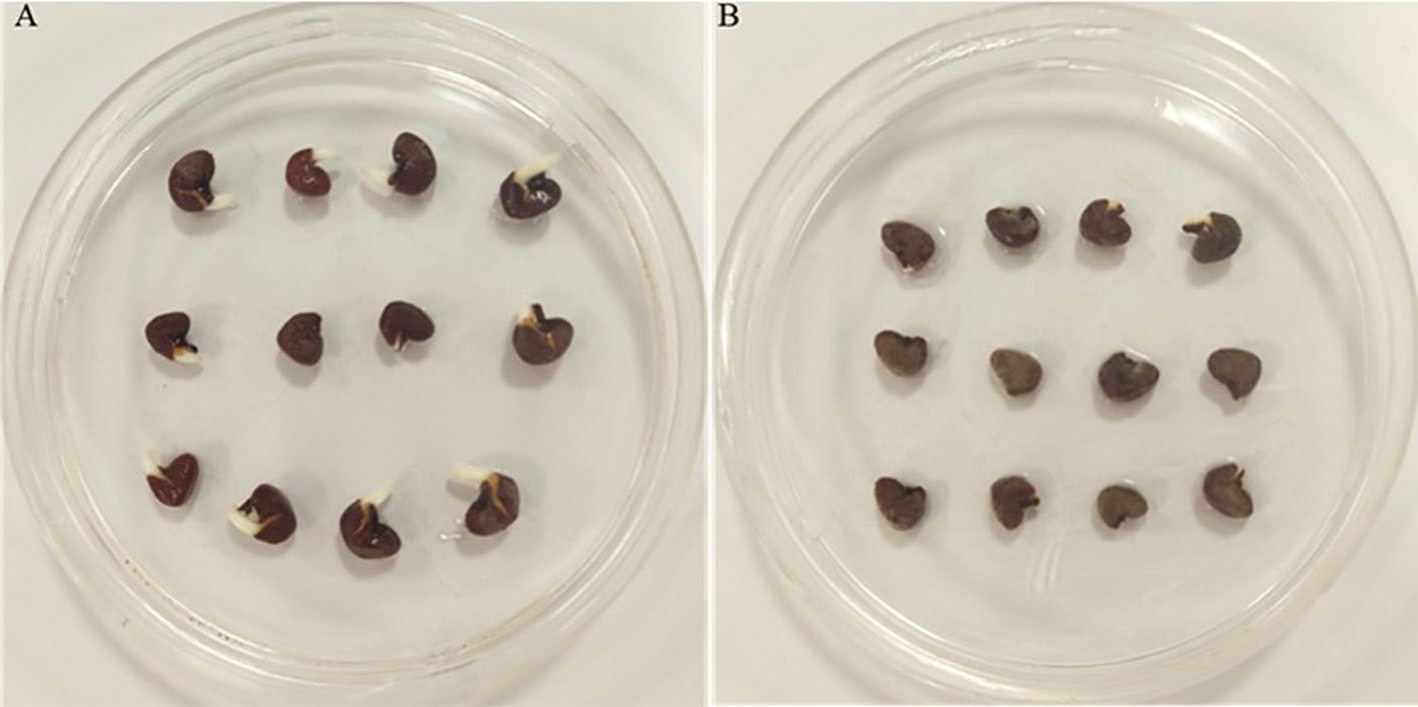
Photographs of noninfected **(A)** and infected seeds **(B)** by *Fusarium solani*.

**Figure 2:**
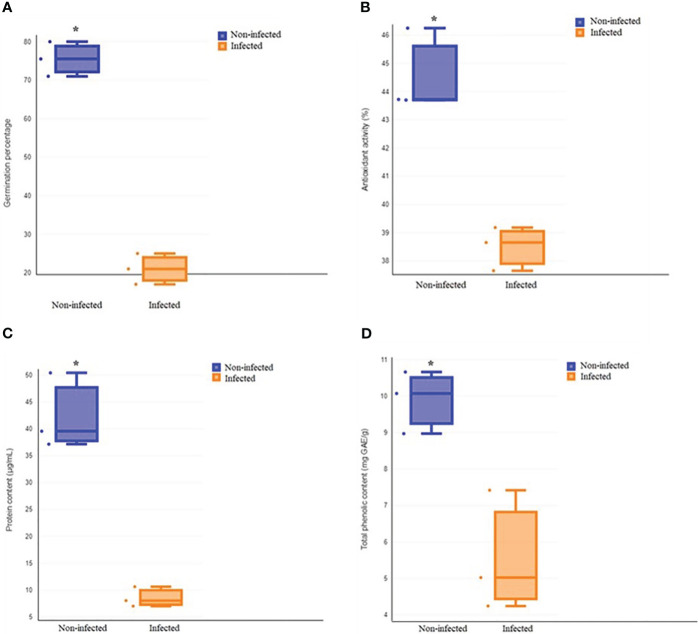
Comparison of total germination **(A)**, antioxidant activity **(B)**, protein content **(C)** and total phenolic content **(D)** between non-infected and infected treatments. Asterisk indicates a statistically significant effect (t-test at *P* < 0.05).

The total germination of infected roselle seeds was 21%, compared to noninfected seeds that had a total germination of 76% (**Figures 1**, **2A**). Fungal infections with *Alternaria alternata* have been shown to hinder the germination of carrot seeds (Zhang et al., 2020), while seed-borne fungi decrease the germination, growth, and yield characteristics of beans (Marcenaro and Valkonen, 2016). Also, different species of *Aspergillus* reduced seed germination (Ibraheem et al., 1987; Garuba et al., 2014), and *Penicillium chrysogenum* decreased the germination of maize seeds (Garuba et al., 2014). In another study, the effect of different fungal isolates was tested on seed germination, and the findings highlighted that some *Fusarium* spp. could remarkably reduce seed germination (Kirkpatrick and Bazzaz, 1979). The inhibition of seed germination might be attributed to the toxic metabolites of fungi (Tylkowska et al., 2003; Garuba et al., 2014). However, seed germination is a highly regulated phenomenon that involves the coordination of many physiological and biochemical processes (Parihar et al., 2015; Tuan et al., 2019). Metabolic pathways involving sugars, amino acids, lipids, and antioxidant defenses are integral to seed germination (Guo et al., 2021; Liu et al., 2021), which may be altered by fungal infection and help explain the effect on seed germination, as described below. It has also been shown that changes in seed reserve composition can influence seed germination (Zhao et al., 2018). In addition, seed imbibition could activate a variety of germination-related biochemical processes that may result in energy reserve hydrolysis into simple forms for embryo uptake (Ali and Elozeiri, 2017). However, the findings of another study revealed that protein and fat reserves were not significantly correlated with seed germination, while soluble sugar and protein contents were positively related to seed germination in six species (Zhao et al., 2018).

### 3.2 Total phenolic content and antioxidant activity

It has been demonstrated that phenolic acids play a key role in seed development and germination and also contribute to the protection of seeds against pathogens (Granger et al., 2011; Corso et al., 2020). Our results showed that fungal infection decreased the antioxidant activity and total phenolic content of roselle seeds (**Figures 2B, D**). Our findings are in agreement with previous studies, which demonstrated that brown spot infection of rice caused by *Helminthosporium oryzae* as a seed-borne fungus decreased plant phenolic content and suppressed the phenol metabolism by producing toxins that aided fungal colonization (Pandey, 2018). Also, infection with *Sclerotium rolfsii* decreased the phenolic content in *Arachis hypogea* (Cardoza et al., 2003). In plants, the defense function of phenolic compounds is partially attributed to their antioxidant properties (Lattanzio, 2013; Krõl et al., 2014). A significant correlation between the total phenolic content and antioxidant activity of plants has been found (Cai et al., 2004; Djeridane et al., 2006). Therefore, the reduction of the phenolic contents of roselle seeds with high levels of infection could have decreased antioxidant activity and weakened the defenses of infected seeds.

### 3.3 Total protein and free amino acid content

**Figure 3:**
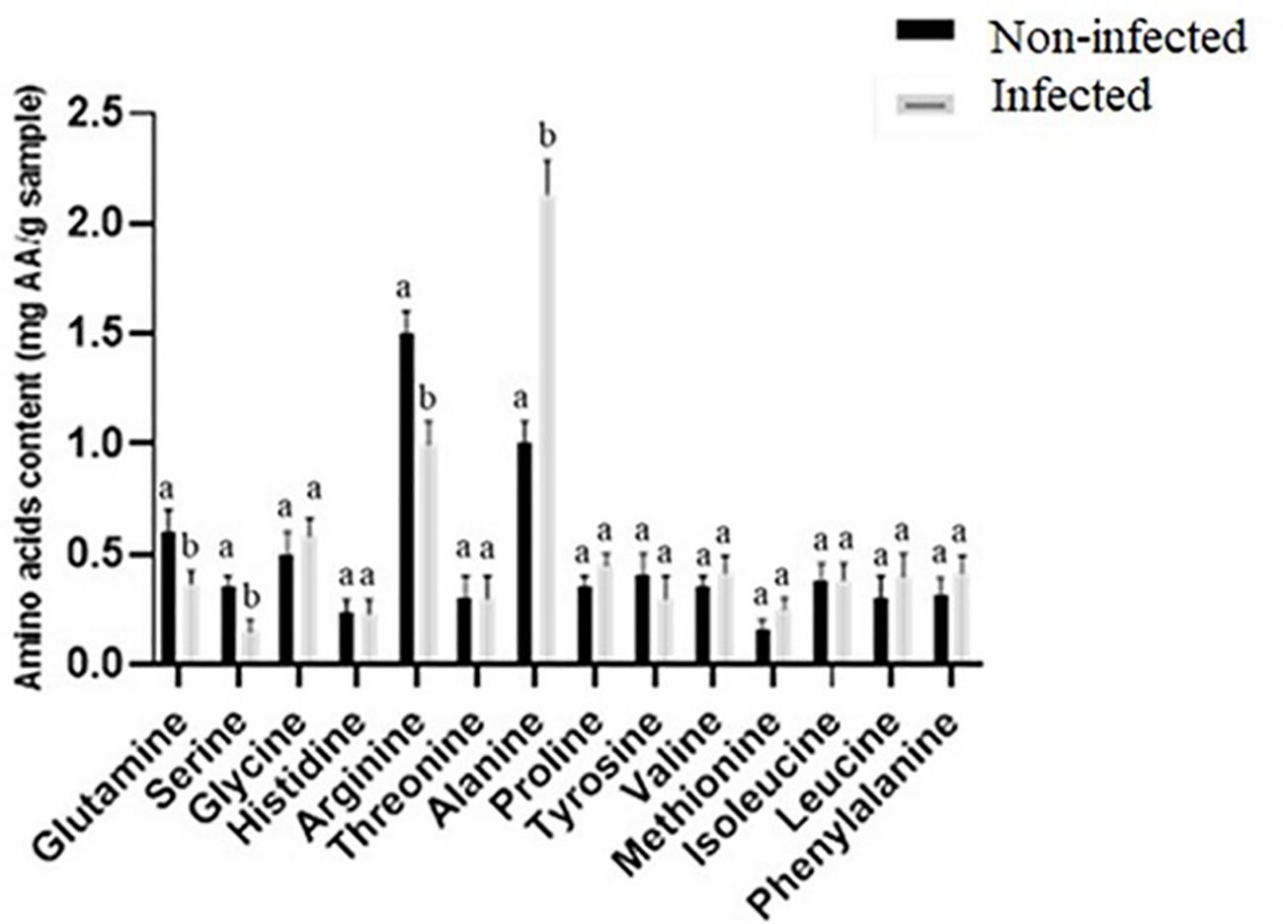
Comparison of amino acid content (mg AA g^−1^ sample) between noninfected and infected seeds. Different letters present a significant difference in each amino acid between noninfected and infected seeds, while similar letters indicate nonsignificant differences (*t*-test at *p*< 0.05).

Amino acids typically accumulate during seed germination (Gerna et al., 2018), in agreement with higher levels of glutamine, serine, and arginine in noninfected seeds (**Figures 2C**, **3**). The expression of glutamine synthetase is upregulated during seed germination in plants (Guan et al., 2015), which agrees with higher glutamine contents in noninfected seeds with higher total germination. Serine contributes to the plant’s response to stress and the formation of the phospholipids required for signal transduction in plants (Waditee et al., 2007; Kishor et al., 2020). Additionally, arginine plays an important role in plant tolerance to stress and contributes to various cellular, physiological, and developmental processes (Matysiak et al., 2020; Hussein et al., 2022). Arginine is also the precursor of nitric oxide and polyamine biosynthesis, which act as key messengers in growth, development, biochemical and physiological processes, and plant adaptation to stress (Yang and Gao, 2007). Amino acid metabolism provides defense compounds, nutrients, and signaling molecules during plant–microbe interaction (Moormann et al., 2022). In contrast to noninfected seeds having generally higher amino acid contents, infected seeds had significantly higher alanine concentrations (2.13-fold) compared to noninfected seeds. Plant-secreted proteins are essential to the plant’s defense against pathogens (Gupta et al., 2015). Our results are similar to studies that showed biotic and abiotic stresses elevate alanine content in plants (Kaplan et al., 2004; Rizhsky et al., 2004; Broeckling et al., 2005; Parthasarathy et al., 2019). Alanine is a defense compound that acts to protect plants from biotic and abiotic stresses (Parthasarathy et al., 2019) and may help seeds fight fungal infection.

### 3.4 Fatty acid content

In the present study, nine FAs were found in the noninfected and infected roselle seeds, with linoleic, oleic, and palmitic acids constituting the major fatty acids in roselle seeds (**Table 1**). The results showed that the concentration of palmitic and heptadecanoic acids was higher (1.13- and 1.4-fold) in infected seeds, whereas linoleic acid was elevated (1.17-fold) in the noninfected seeds compared to infected ones (**Table 1**). Fatty acid metabolic pathways contribute to plant defense against pathogens (Kachroo and Kachroo, 2009). Changes in fatty acid composition might influence the seed’s response to the fungal infection. However, fungal infection employs different strategies to subvert plant defense responses by altering plant physiology, which results in fungal colonization of seeds. They use these strategies to hijack, evade, or counteract plant defense responses to complete the life cycle and produce viable progeny. The main fungal strategies for inhibiting plant defense responses include subverting reactive oxygen species (ROS) damage, manipulating tissue pH, inhibiting host proteases, and subverting hormone signaling (Rodriguez-Moreno et al., 2018).

**Table 1:** Fatty acid composition (%) of noninfected and infected treatments.

	SFA (%)					UFA (%)			
Treatment	Myristic acid (C14:0)	Palmitic acid (C16:0)	Heptadecanoic acid (C17:0)	Stearic acid (C18:0)	Arachidic acid (C20:0)	Palmitoleic acid (C16:1)	Oleic acid (C18:1)	Linoleic acid (C18:2)	Linolenic acid (C18:3)
Noninfected seeds	0.50^a^ ± 0.30	25.85^a^ ± 0.65	0.10^a^ ± 0.01	1.04^a^ ± 0.09	0.75^a^ ± 0.07	0.58^a^ ± 0.04	28.90^a^ ± 0.38	40.40^a^ ± 0.60	1.87^a^ ± 0.50
Infected seeds	0.73^a^ ± 0.41	29.13^b^ ± 1.49	0.14^b^ ± 0.01	1.18^a^ ± 0.10	0.73^a^ ± 0.31	0.46^a^ ± 0.11	31.55^a^ ± 1.65	34.54^b^ ± 2.73	1.53^a^ ± 0.07

SFA, saturated fatty acid; UFA, unsaturated fatty acid. C14:0, C16:0, C17:0, C18:0, C20:0, C16:1, C18:1, C18:2, and C18:3 indicate lipid numbers as a numerical representation of fatty acids. Values are expressed as the mean of three replicates ± SD. Values in each column with different letters are significantly different (t-test at p < 0.05) between each fatty acid.

Saturated fatty acids were relatively higher (1.13-fold) in the infected roselle seeds, while unsaturated fatty acids were relatively higher (1.05-fold) in the noninfected seeds. Thus, the ratio of unsaturated to saturated fatty acids was significantly different when comparing noninfected and infected seeds (2.54- and 2.13-fold, respectively). This is potentially related to the seed–fungus interaction leading to oxidative bursts of ROS oxidizing the polyunsaturated fatty acids (e.g., linolenic and linoleic acid). In turn, this would influence the quality of seed oil.

### 3.5 Sugar contents

**Figure 4:**
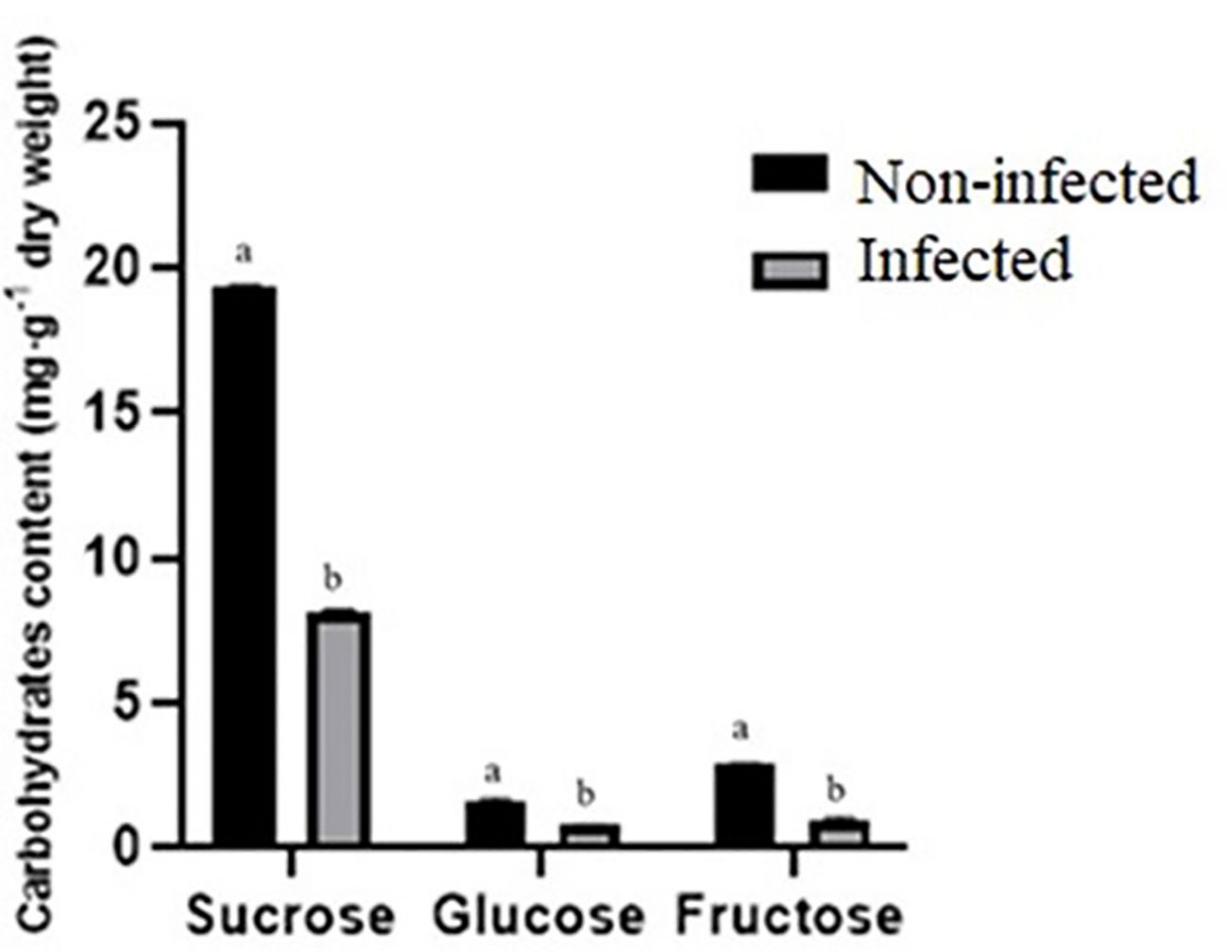
Comparison of carbohydrate content (mg g^−1^ of dry weight) between noninfected and infected seeds. Different letters present a significant difference in each amino acid between noninfected and infected treatments, while similar letters indicate nonsignificant differences (*t*-test at *p *< 0.05).

Sucrose, fructose, and glucose contents were lower in infected roselle seeds compared to noninfected seeds (**Figure 4**). Sugars play a key function in plant defense responses and the immune system’s defense against fungal pathogens (Morkunas and Ratajczak, 2014). Fungi reduce the level of sugars through their uptake and consumption for energy and structural purposes (Morkunas and Ratajczak, 2014). Our results are in agreement with another study, which showed that the infection of *Sclerotinia sclerotiorum* reduced the levels of sucrose, fructose, and glucose during cotyledon infection of sunflower (Jobic et al., 2007). In addition, the sugar content decreased during tomato–*Botrytis cinerea* interaction (Berger et al., 2004). It was also demonstrated that sporulation of *Puccinia triticina* showed a competitive priority for assimilation in wheat (Bancal et al., 2012). The degradation of storage reserves is vital for the germination of mature seeds, and the sugars obtained from the hydrolysis of starch are the main energy source for seedling emergence (Beck and Ziegler, 1989). It has also been shown that carbohydrates and proteins are required to provide energy and substrates for the seed germination of *Polygonatum cyrtonema* (Liu et al., 2021). Therefore, the low germination in infected roselle seeds may be explained by the decreased availability of sugars and proteins.

## 4 Conclusion

In this study, the chemical composition and energy reserves of roselle seeds were analyzed during *F. solani* infection. *F. solani* infection deteriorated the quality of roselle seeds, reduced total germination, and altered the chemical composition, including energy reserves of seeds. The biochemical changes, such as lowered sugar and amino acid availability, induced by the fungal infection could interfere with the requirements of the seed during germination, leading to lowered seed vigor and less germination. In this study, the fungal infection decreased antioxidant activity, total phenolic content, protein, sugar, and some amino acid (glutamine, serine, arginine) contents. Therefore, these changes in the chemical composition of roselle seeds caused by *F. solani* infection may negatively influence the germination of roselle seeds.

## Data availability statement

The original contributions presented in the study are publicly available. This data can be found here: Figshare, https://figshare.com/, doi: 10.6084/m9.figshare.23244140 and doi: 10.6084/m9.figshare.23244137.

## Author contributions

AT designed and performed the experiments. AT, TR, SYS, and CWL wrote and edited the article. All authors contributed to the article and approved the submitted version.
